# Extracts and Gleanings

**Published:** 1871-04

**Authors:** 


### PART II.

### Abridged Extracts and Gleanings from our Exchanges

*MULTUM IN PARVO.*

Skin-Grafting.—Dr. David Page, of Edinburgh, has made some investigations (British Medical Journal, Dec. 17, 1870,) which throw a very important and interesting light on this subject. All observers agree in describing the disappearance of the characters of true skin in the piece laid on the wound, and the resulting appearance of a small pellicle, which ultimately becomes a centre of cicatrization. Mr. Nelson Dobson, in the paper which he read at the Bath and Bristol Branch of the British Medical Association, described the excellent results from splitting fine shavings of skin into small pieces; and all authorities have agreed from the first that the skin taken should be completely free from subjacent areolar or adipose tissue. Dr. Page goes still further. Microscopic examinations into the physiological characters of the process have convinced him that "the so-called skin-grafting consists in truth not of a transplantation of true skin, but of epithelium." The pellicle, which retained its vitality, and proved of initial value in inducing cicatrization, was found to consist of young epithelial cells. Further, the cicatrix thus induced, and that formed by the ordinary normal process elsewhere, were undistinguishable one from the other, and, under the microscope, proved to be of the same fibro-cellular character.

Dr. David Fiddes, of the Royal Infirmary, Aberdeen, has clinically arrived at a conclusion which affords a remarkable support of Dr. Page's observations and deductions, if confirmed by further observations. He says that it is quite unnecessary to put the patient to the pain of cutting a piece of healthy skin from the body for the purpose of transplanting it on the sore. "All that is necessary to be done," he says, "is to take a long bistoury or razor, and shave or scrape off the epidermic scales from the convex parts of the extremities, such as the outer and convex aspects of the forearms and thighs, and place them on the healthy granulations. This can best be done by brushing the scales off the bistoury with a camel-hair pencil. After securing them *in situ* for three or four days by means of common adhesive plaster, the ganulations on which the epidermic scales were placed assume a glazed bluish appearance, which gradually grows into skin, and meets the nearest edge of the healing ulcer, which edge shoots out and meets the newly-formed skin on the granulations. The result is continuity of healthy skin." This last, it will be observed, is a very important statement. Dr. Page's observations lead him to the opposite conclusion, that there is no nearer approach to the condition of true skin than in cases of ordinary cicatrization, but only a more rapid formation, and from a greater number of centres, of a tissue of very low vitality, deficient in many of the functions of the true skin, and easily destroyed by causes that would not affect the latter. The sphere of usefulness of the operation will be largely affected, according as the one or other view proves to be correct. No doubt clinical observations will multiply; and we direct attention to these points, as the best method of furthering the discovery of the place in surgery of this new and suggestive practice.—*British Medical Journal*, Dec. 24, 1870.

Bromide of Potassium in Ague.—Bromide of potassium was first introduced into medical practice, as a remedy for enlargement of the spleen, by Dr. Williams. Dr. Moxon has lately been trying among the out-patients at Guy's Hospital, the bromide in cases of ague (British Med. Journ., June 11, 1860.) The results are such as to show that this drug possesses very remarkable power over ague, and a power that promises to be of important use in many of the more obstinate cases. In one case illustrative of its action, the patient had had tertian ague for two months before admission to the hospital, and had taken quinia without benefit; after admission, he took eighty-four grains more without benefit. Twenty-grain doses of bromide, three times a day, were then given, and he was discharged cured in ten days. It is found always to check the ague, and often to cure it permanently, not always; but that is the case with any remedy.—*Med. News and Library.*

Sulphurous Acid in Typhoid Fever.—Dr. Wilkes, of Ashford, Kent, affirms (Med. Press and Circular, Dec. 14, 1870,) that he has had very great success in the treatment of typhoid fever by means of small doses of sulphurous acid. He gives the acid with syrup of oranges in water, in doses of from two and a half to twenty minims every four hours, until the patient complains of tasting, as it were, lucifer matches. He alleges that this remedy arrests the further development of the fever-poison, and thus *exterminates* the fever. "Briefly, it is an antidote." Of 173 cases of typhoid fever treated, only two died, and these two did not take the acid.—*Med. News and Library.*

CALABAR BEAN IN CONSTIPATION.—Dr. Victor Subbotin (Arch. f. Klin. Med., vi., 2, 3, 1869,) communicates cases in which he obtained remarkably good results from this remedy. He prescribed a solution of the extract in glycerine, one to thirty, the dose being four drops four times daily. A fecal tumour which resisted strong doses of a cathartic was quickly dispelled in this way. The cases in which the treatment is most suitable, are those due to atony of the muscular coat of the bowels, on which the calabar extract acts powerfully, as is shown by experiments on animals.—*Med. Press and Circular*, Jan. 4, 1871.

ETIOLOGY AND TREATMENT OF DIPTHERIA.—Prof. Steiner, of Prague, has published in the Jahr. f. Kinderk, of November 1st, 1870, a series of cases in which he was able to try different kinds of treatment, and watch the result. He thinks that the etiological question is not as yet solved, although very great authorities consider diphtheria to depend on blood-poisoning. Nor can the purely local cause, in the shape of germs, be satisfactorily proved. Further investigations are indispensable to decide between the two theories. Therapeutically, the professor considers that the means which answered best in his hands are lime-water locally, and, internally, quinia, chlorate of potassa, and wine ; when laryngitis occurs, emetics ; and if they act no more, tracheotomy. When paralysis follows the acute attack, tonics are to be recommended, but the author has found that in many cases the symptoms of paralysis disappeared without treatment.

Dr. Moritz Schlier has tried carbolic acid extensively in diphtheria, his reason for using it being the supposed existence of germs in this disease. The solution used was 1 in 16 and it was energetically applied to the false membrane by means of the finger wrapped in some linen. No internal remedy was employed ; but those patients whose age allowed them to gargle, were made to wash out the mouth with the above-named solution every half hour. Out of 36 cases, 13 recovered, and these were all very severe ; most of the patients, also, were out of danger in about four or five days. During convalesence, tonics and wine were liberally used.—*Lancet*, Jan. 7, 1871.

ANÆMIA, LEUCHORRHŒA.—Mr. J. C. Dickerson regards *lactate of iron* as a tonic and blood-restorer of great value, especially in those numerous female complaints associated with great debility, such as anæmia, chloro-anæmia, leucorrhœa and passive hemorrhage. In functional palpitation of the heart dependant upon debility, and in certain forms of dyspepsia, it may often be prescribed with advantage.—*Braithwaite's Retro.*

ARSENIC.—There are few remedies more useful in many diseases of the stomach than arsenic. In the so-called irritative dyspepsia, where the tongue is furred and its papillæ red and prominent, a drop of the solution of arsenic taken shortly before food will be found of great benefit. It is almost unfailing, when administered in the same quantity and at the same time, in the vomiting of drunkards. This usually occurs in the morning before breakfast. It is accompanied by great straining and distress, but generally very little, and sometimes nothing, is ejected. In the latter case it is called dry vomiting. The vomited matters are generally intensely bitter and sour, and of a green color. To arrest this symptom there is no remedy like arsenic. In chronic ulcer, and even in cancer of the stomach, arsenic is invaluable, by alleviating the pain and checking the vomiting of these complaints. Small doses of arsenic generally benefit persons with whom the food simply regurgitates, and is rejected without effort. A solution of arsenic in one or two drop doses is always of service in that form of chronic dyspepsia and diarrhœa called lientery, and which is most common in children from eight to twelve years of age. It does good almost immediately, and generally effects a cure in a week or ten days.

Arsenic should never be given on an empty stomach, nor in increasing doses. Children about five years old will bear nearly as large a dose as grown-up people. The largest dose ever required is (of the solution,) five drops, repeated three times a day. An exception to the last rule is furnished by chorea, where, if the affection has resisted this quantity, the dose may be cautiously augmented.—*American Practitioner.*

SLOUGHING SYPHILITIC SORE THROAT.—Dr. C. S. Smith (Lancet,) derives great benefit in such cases by painting over the diseased surface with Calvert's liquid carbolic acid undiluted, by means of a camel-hair pencil. The pencil must be pushed well upwards and downwards so as to reach, as much as possible, every part of the diseased surface. A weaker solution should afterwards be employed frequently. Good nourishment should be given with the following mixture.

| | | |
|---|---|---|
| ℞—Liquor cinchonæ, | m. v. | |
| Batley's solut. of opium, | m. v. | |
| Iodide potassium, | grs. x. | |
| Water, | ℥ i. | m. |

S. This quantity three times a day.

CHRONIC CATARRH.—The tincture of aconite in five-drop doses every four hours has cured this troublesome symptom when the ordinary remedies have failed.—*Medical Archives.*

TREATMENT OF GONORRHŒA BY LOCAL MEANS.—Dr. Thomas Hill, of Kennansville, North Carolina, writes to the Richmond and Louisville Medical Journal:

The very first case of this disease I was called to treat, the question presented itself to my mind, why, if this is a local disease, should I treat it constitutionally? Why fill the stomach with such nauseous drugs as copaiba, cubebs and all such gums? Why may not this inflammation, purely local in its character, be treated as a simple local inflammation? Then if the specific disease still persists, if these remedies have a specific effect, why not apply them directly to the disease? I was not long in making up my mind to test the accuracy of my reasonings. I ordered the patient to retire at once to bed; gave him a syringe and directed that he should inject cold water into the urethra every half hour during the day, and to keep a cloth wet with cold water applied all the time to the parts. The next morning, on visiting him, I found the inflammation almost gone, and the discharge much thinner and of a lighter color. I had in the meantime prepared a bottle of Chapman's mixture, which I then directed him to inject once every four hours, the cold water to be continued. This treatment was continued for two days, when the case was, to all appearance cured; to make assurance doubly sure, though, he was directed to use the mixture twice a day, for a few days. He had no further trouble.

For the last fifteen years this has been my practice, sometimes using the copaiba,.at others, sulph. zinci, tannin, etc. During the last twelve months, having witnessed the admirable effect of carbolic acid in the treatment of local inflammation and destroying specific virus, I determined to try its effects in gonorrhœa. After subduing the inflammation with cold water, I prepared the following:

| | | |
|---|---|---|
| ℞. | Acid carbolic, . . . | gtt. v to x. |
| | Glycerin, | |
| | Water, . . . | aa. ℥ ss. m. |

This to be used three times in the twenty-four hours. I have yet to see the patient, who followed these directions, who was not entirely relieved in three days. I look upon rest in the recumbent position, as absolutely requisite. If any internal remedy is necessary, a good dose of epsom salts will be found the best. If the scalding of the urine be very severe, a teaspoonful of bicarb. soda in a tumbler of water, twice a day, will be found very effectual.

I have never seen any injurious results follow from the above treatment—such as stricture, swelled testicle, etc.

Treatment of Diarrhœa.—Dr. G. M. Noble recommends the following treatment:

Soften some resin by melting it with a little lard. Make it soft enough to form it into pills. Then take forty-eight grains of the resin and four grains of nit. silver finely pulverized, mix intimately, and make eight pills. Give them once in eight hours. Give them when the stomach is empty.

I have used these pills a long time and do not remember a case in which they failed of producing a cure.

They have proved also an excellent remedy for dysentery. The resin will not dissolve in the stomach, but will dissolve in the intestines. A six grain pill of this resin will dissolve while passing through the alimentary canal. The nitrate of silver in this way is conveyed chemically unchanged to the part that is diseased. If it is given according to the usual methods, it undergoes a chemical change, and is not nitrate of silver when it reaches the afflicted part.—*Med. and Surg. Reporter.*

Hydrangea Aborescens.—We have frequently called attention to this plant as a remedy for the removal of sabulous and gravelly deposits from the bladder. It has also been useful in other affections of the bladder.

A correspondent in Alabama, Dr. John H. Parrish, writes:

"Since the year 1853, I have occasionally prescribed hydrangea aborescens in my practice, and with very satisfactory results. I have occasionally had a preparation that proved utterly worthless, while another preparation would prove very useful to the same patient. Is this difference in the effect of the medicine to be attributed to the mode of preparing, the season in which the plant is gathered, or to what? When should the plant be gathered? How treated? What the best mode of preparing? Should any part but the root be used?

Reply.—The bark of the root is the part we originally recommended. The best time to gather the roots is in early fall, or just before the flowers mature. The fluid extract now extensively manufrctured is, we believe, a reliable preparation, but we should prefer a syrup made in the following manner:

| | | |
|---|---|---|
| ℞—Fresh root of hydrangea, | . . | 2 lbs. |
| Water, | . . | 6 qts. |

Boil down to two quarts, strain and add one quart of honey, and boil down to one quart. Dose—A teaspoouful two or three times daily. An overdose has produced vertigo.—*Dr. S. W. Butler—Med. and Surg. Reporter.*

Choral in Puerperal Convulsions.—Dr. R. D. Fox reports a case in which he gave ʒ ss choral. The convulsions which had before been frequent immediately ceased.

CREOSOTE.—The author believes that the failure of the remedy to check nausea and retching in many cases is owing to the largeness of the dose that is given, and which often itself produces the very symptoms for which it is administered. Its best effects are obtained if just sufficient creosote is added to water to make it taste distinctly but not strongly of the medicine, and of this a dessert-spoonful may be taken frequently. Thus employed, this remedy will often prove effectual in staying nausea and retching, but is considered by some persons to have less power over actual vomiting.—*American Practitioner.*

MALARIAL FEVER.—The following is a favorite formula:

℞—Ext. colocynth. co.,

Quiniæ sulphatis, . . . aa. gr. xij.

Ext. ignatiæ alc.,

Piperine, . . . aa. gr. vj.

Morphiæ sulph. . . . gr. j. M.

Ft. in pil. no. xij. div.

Sig.—One every two, three or four hours.

*Med. and Surg. Reporter.*

IRON AND QUINIA APPLIED LOCALLY IN ERYSIPELAS.—Dr. Garretson says that iron and quinia in the following proportion is most effectual in killing the disease:—Take Tinct. ferri chloridii, four fluid ounces, quinæ sulphatis, twenty-five grains. When a wound exhibits the slightest erysipelatous tendency, dilute with two-thirds water, and with a brush, wash the part. If a case is first seen with the type fully developed, dilute with one part water, and use as before; if a change is not seen within an hour, employ the full strength; this on the tender skin will form a blister, but will remove the disease.—*Half-Yearly Comp. of Med. Science.*

PHLEGMASIA DOLENS TREATED BY OPIUM.—Dr. C. C. P. Clark remarks that opium not only relieves the pains of *phlegmasia dolens*, but it is actually curative with great rapidity and certainty. The rule is to keep the pain thoroughly subdued, as the earlier the treatment is commenced, the more effectual it becomes. The first symptom is pain low down in the pelvis—this should be remembered, and the opium given at once.

THE SULPHATE OF COPPER, either by the mouth or as an injection into the rectum, is often effectual in staying severe chronic or acute diarrhœa, whether it depends on organic disease or not. —*American Practitioner.*

On the Treatment of Sciatica.—By Dr. J. Waring-Curran.—[Hypodermic injection of morphia is a wonderful remedy in sciatica, but unfortunately the pain often returns with increased severity as soon as the effects of the morphia pass off. For some years Dr. Curran has employed the following treatment, and he states that it is safer, surer and more satisfactory than any treatment or method of treatment he has anywhere observed.]

In a small porcelain vessel I mix one grain of morphine and three grains of extract of belladonna with six drops of creosote. I get my patient out of bed, standing as erect as the nature of the disease will permit him, and begin making small incisions, half an inch long, with an intervening space of three inches between each incision, cutting only through the skin and subcutaneous cellular tissues. I make the incisions alternate on each side of the nerve, beginning underneath the fold of the *gluteus maximus.* Having wiped off the effused blood, I quickly rub in the composition. The morphine and belladonna allay the pain, and the creosote sets up, if properly applied, a certain amount of local irritation, which is very desirable. M. du Chaillu, in his exhaustive and popular work on the gorilla, records a somewhat similar proceedure existing among the Celond races. If my memory serves me, caustic lime is the agent he records as being employed.

To every patient suffering from sciatica, I exhibit iodide of ammonium, and I have remarked, as I hope soon to show, that its therapeutic power is superior to the iodide of potassium, but in no complaint will this be apprcciated more than in the eruption stages of syphilis and in diseases of the glandular system. The patient bent double with acute pain, will be found after the incisions are made and the morphine composition rubbed in, able to move his leg freely in any direction. There is, of course, a numb feeling experienced, but the liberation from acute suffering provokes an expression of gratitude, which is conclusive evidence of the value of the plan of treatment advocated.—*Med. Press and Circular.*

Chloral to Children.—Chloral may be given with perfect safety and success to children, in doses of two grains to an infant of 18 months; of 3 grains at three years of age, and of 10 grs. at 9 or 14 years.

Anæsthetic Property of Carbolic Acid.—Mr. E. Wilson (Jour. Cutaneous Medicine,) asserts that carbolic acid has a striking anæsthetic power. "I employ it" he writes "at present very commonly, previous to the application of caustic to *lupus and epithelioma.* It benumbs the surface, it dulls the excessive sensibility of the superficial nerves, and it thereby permits the caustic application of our remedies with a great reduction in the amount of pain."

Apium Graveolens in Amenorrhœa.—Prof. Pietro Gamberini, Surgeon-in-Chief of the Hospital of St. Oriola, writes in the *Bulletin of Medical Sciences* of Bologna, of November, 1870, that he has found that the essential oil of *apium graveolens* (garden celery.) contains highly emmenagogue properties; he finds it to answer very well in some cases of obstinate *amenorrhœa*, where all other remedies in common use have failed. He gives it in doses of twenty to thirty drops, two or three times daily.

Carbolic Acid in Carbuncle.—Dr. J. C. Nott, of New York, (New York Medical Journal,) advocates the local use of carbolic acid in carbuncle, and publishes an obstinate case which was promptly cured after seven applications. He believes this so-called acid to be more penetrating in its action, more efficient as an antiseptic, than subsulphate of iron, advocated by Dr. Blake, of this city, in the treatment of fibrous tumors of the uterus.

Chlorate of Potassa.—Dr. Simpson first suggested the use of chlorate of potassa as a preventive of abortion, on the ground that it would add oxygen to the system, for the restoration and arterialization of blood.

Fordyce Barker says: "Whether the theory be correct or not, the clinical fact of its value I am thoroughly convinced of." The administration of the medicine in those who have had habitual abortion, has been to increase the motions of the fœtus, and such remarked the feebleness or absence of motions when the medicine was suspended.—*Lancet and Observer.*

Digitalis in Orchitis.—In the Bulletin de Therapeutique, for February, 1870, Dr. Besnier states that having frequently seen M. Dibout employ digitalis with success as an outward application in cases of hydrocele, he has adopted the same plan in cases of orchitis, however produced, and the results obtained have been such as to warrant further trial. The mode of procedure he adopted was the following: The invalid is kept at perfect rest, with the scrotum conveniently raised, and constantly surrounded with compresses soaked in a concentrated effusion of the leaves of the digitalis. The infusion may be either warm or cold, as most agreeable to the feelings of the patient, but under any circumstances they should be kept perfectly moist; a covering of oiled silk being placed over all.—*Half-Yearly Compendium of Medical Sieence*, July, 1870.

Management of Pertussis.—In Newark, N. J., most of the physicians send their patients to the purifying room of the gas works, to spend an hour or two daily, the inhalation of the fumes arising from the gas passing through lime having the effect of moderating and shortening the disease. In cases where it is inconvenient to visit the works, carbonate of lime has been produced therefrom, and placed in vessels about the room in quantities sufficient to impregnate the atmosphere, and has been attended with good results. When access to gas works cannot be had, the hydro phenil, which, when unpurified, is called benzine, is recommended by Braithwaite ; and the same results obtained as from a residence in the purifying chambers of the gas works. Ten or fifteen drops are given in a little water to a child every day, and as soon as it is asleep a few drops are sprinkled on the pillow, so that the smell is diffused throughout the room.—*Trans. Med. Soc. of N. J.—Oregon Medical and Surgical Reporter.*

Bromide of Potassium in Dentition.—Dr. Salvatore Caro, in the Medical Record, strongly recommends the local application of this remedy to allay irritation of the gums in dentition. He says : "In the most severe cases of odontitis, either with or withont ulcerated gums and loose bowels, I have never failed to relieve the child by the application of the bromide of potassium. Almost immediately after the first rubbing of the gums, from being swollen, turgid and red, they assume their natural color, and a certain amount of ease is felt. Saliva commences to dribble ; and, as if by enchantment, agitation' carpopedal involuntary motion, vomiting and looseness of the bowels disappear. As the vomiting and diarrhea in this case are not the consequence of gastro enteritis, but of an excitement of the stomach and the intestinal mucous membrane, owing to the inflamed condition of the gums, I suppose it will never be cured, either by scarification of the gums, or by the use of astringents or anodynes; but as I shall hereafter prove, simply by the use of bromide of potassium."—*Dental Cosmos.—Oregon Medical and Surgical Reporter.*

Liquor Calcis in Albuminuria.—A German practitioner recommends lime water as a diuretic in acute Bright's Disease and general anasarca. He used it with success in one case, the urine gradnally increasing, the albumen diminishing, and the tube casts inceasing.

Hemorrhoids.—Dr. A. L. Hudson says, in the Pacific Medical and Surgical Journal that dry hyd. chlor. mitis applied once or twice a day to tumid and tender hemorrhoids situated about the anus, rarely fails to *cure* them in a few days.

A FAVORITE TONIC REMEDY—Which makes an eligible preparation, and one very palatable, is as follows:

℞—Acidi phosphorici dil. . . ℥ i.

Elix. calisay, . . ℥ iv.

Elix. valer ammon., . . ℥ ii.

Glycerinæ, . . ℥ iii.

Sherry wine, . . ℥ vi. m.

Dose from one half to one ounce.

"It is from an experience in the use of this remedy in more than two hundred cases, extending over a period of several years, that confidence has been inspired in its general adaptation to the treatment of diseases marked by debility of the nervous system. *Cincinnati Medical Repertory*, March, 1870.

TREATMENT IN DIARRHEA IN INFANTS.—Dr. Smith (Medical News and Library,) in his valuable papers on the "Wasting Diseases of Infants and Children," recommends the following prescription, if the bowels are rather loose, with dark, slimy, offensive stools:

℞—Tinct opii, . . . . ♏ viii.

Ol. ricini, . . . . ʒ j.

Syrupi zingib

Mucilag. Acaciæ, . . . aa ℥ j.

M. S. A teaspoonful three times daily. In the screaming fits, accompanied by constipation, this combination of castor-oil with laudanum is very valuable.—*Medical Record.—Druggists' Circular and Chemical Gazette.*

UNGENT FOR BRONCHOCELE.—Prof. James R. Wood, of New York, extols the following formula as an ointment in bronchocele and other glandular tumors:

℞—Ung. stramonii, . . . ℥ ij.

Ext. conii, . . . ʒ ij.

Iodid. potassii, . . . ʒ ij.

Iodidi, . . . gr. x.

—*Detroit Review of Medicine and Pharmacy*, July 1869, p. 393.

CARB. AMMONIA IN PNEUMONITIS.—Dr. A. Patton, of Vincennes, Ind., reports in the American Journal of Medical Sciences, for October, 309 cases of severe and well-marked pneumonitis treated by himself and others with carb. of ammonia of which only 8 died, or 1 in 28, cases. He gives 5 to ten grains every two hours, night and day, from the commencement of the attack. In some instances blisters and quinia were also employed.—*Pacific Medical and Surgical Journal*, Dec. 1870.

Hydrate of Chloral.—Prof. Samuel G. Armor, M. D. of Long Island College Hospital (Mich Univ. Med. Journal,) arrives at the following conclusions as to the physiological and therapeutic action of this new drug. It cannot always be relied on as a substitute for many of the older and well-tried anodynes and nervines.

In a certain proportion of cases it produces unpleasant symptoms, such as gastric distress, difficult breathing, partial paralysis of the organs of deglutition, and a restless condition of the brain and nervous system. This is, however, largely exceptional to its general action.

These unpleasant symptoms are in many cases obviated by combining an opiate, in small sustaining doses, to the nervous system with chloral—say the 1-12th of a grain of morphine, or 10 or 12 minims of McMunn's elixir of opium (he prefers to administer the opium separately). The action of the opium thus administered appears to be antagonistic to the sometimes depressing effects of the chloral.

Chloral should never be administered on a full stomach, neither on an empty one; intermediate periods are better. A good rule is to select a period when the stomach is empty, and have the patient take a small crust of bread or a cracker ten or twelve minutes before taking the chloral. Its action is somewhat transient. In two or two and a half hours the dose must be repeated if the first produces no effect, or if we desire to protract the action of the drug.

The protracted use of the drug is not advisable. It weakens the general vital forces, and tends to the production of anæmia.

Gelseminum Sempervirens.—Dr. E. P. Hurd, Newburyport, Mass. (Boston Medical and Surgical Journal), has been in the habit for some time of using a tincture of the root of the yellow jessamine, and believes that as a cardiac sedative we have not its equal in the whole range of the materia medica. It relieves in a marked manner the shortness of breath and palpitations of all forms of heart disease. He has seen more prompt and decided benefit from its use in chronic valvular disease than from digitalis. The dose may be three drops of the saturated tincture every two, three or four hours. The gelseminum is combined with Hoffman's anodyne and tincture of lavender, and is believed to have a specific effect on the vaso-motor nerves, stimulating them, and thus equalizing the circulation and lessening the labor of the heart. It also allays the nervous irritability, is surer than veratrum or prussic acid, and safer than digitalis.

Diphtheria was treated by the late Dr. Magruder, of Washington, as it is claimed, with most marked success, on the following plan :

The throat was rubbed frequently, externally, as soon as the swelling and soreness commenced, with spirits of turpentine. Internally, this :

| | |
|---|---|
| ℞—Potassæ chlorat. . . . | ʒ j. |
| Tinct. guaica comp. . . . | ʒ ij. |
| Tinct. cinchonæ, . . . | ʒ ij. |
| Mellis, . . . | ℥ ss. |
| Aq., . . . | ℥ iijss. |

M.—S. A tablespoonful every three hours. Twenty drops of the muriated tinct. iron, an hour and a half after each dose.

It is said that 82 out of 85 cases were successfully treated.—*Chicago Medical Journal*, November, 1869.

Perchloride of Iron in Urinary Diseases.—Dr. Lionel S. Beale has advocated the use of the aqueous solution of perchloride of iron in diseases of the urinary organs. He believes it to be efficacious as the tincture, while at the same time it is cheaper. Dr. Beale is satisfied of the advantage of the presence of free acid, and in cases of irritable bladder, uses the following combination; in this case, however, substituting the tincture for the aqueous solution :

| | Brit. Pharm. |
|---|---|
| ℞—Tr. ferri perchlor, . . | min. x to ʒ ss. |
| Acid. hydrochlor. dil. . . | min. x to ʒ ss. |
| Tr. hyosciami, . . | min. x to ʒ ss. |
| Sp. chloroform, . . | ʒ ss. |
| Inf. quassiæ ad. . . | ℥ j. to ℥ jss. |

Mix. To be taken twice a day, at 11 and 4 o'clock.—*Druggists' Circular and Chemical Gazette.*

A Good Anæsthetic Mixture.—Dy. Henry F. Lyster, of Detroit (Mich. Univ. Med. Journal), advocates this anæsthetic mixture in private practice :

| | |
|---|---|
| ℞—Alcoholi, . . . . | ℥ ss. |
| Chloroformi, . . . . | ℥ j. |
| Æther. sulphurici, . . | ℥ ij. |

Misce.

Before administering the above in surgical operations, he is in the habit of giving the patient a teaspoonful or two of brandy, thus corroborating the views of Dr. Warren Stone, of New Orleans, and others.

EMBROCATION FOR INFLAMMATION OF BREASTS.

| | |
|---|---|
| ℞—Fluid extract of aconite, . | One half ounce. |
| " " phytolacca, | One ounce. |
| Iodide poatassium, | One drachm. |
| Warm water, | One pint. |

Wet linen compresses, and apply constantly.

No application with which I am acquainted will so surely prevent tumors of the mammæ from taking on the suppuration process. Even if suppuration have taken place, it resolves the indurations and relieves pain.—*Med. Bulletin.*—*Chicago Med. Jour.* January, 1871.

SULPHITE OF SODA IN CHRONIC CYSTITIS.—Mr. L. Wilcox, late house surgeon of King's College Hospital, recommends the use of sulphites in those cases of chronic crystitis where the urine decomposes before it is eliminated. He finds that by the employment of sulphite all the putridity disappears, and the urine becomes clear and colorless.—*The Practitioner.*—*Med. News.*—*Medical Archives.*

BROMIDE OF POTASSIUM IN LEUCORRHŒA.—Dr. A. H. Kinnear reports (Chicago Medical Journal,) twelve well-marked cases of leucorrhœa, none of which were of less than six months' standing, where bromide of potassium was administered with excellent success in doses of grs. xx twice a day. Under this treatment the disease yielded in a majority of cases in one month. He also gave the remedy in a case of gleet, which resulted in a perfect cure in the course of a week.

OBSTINATE HICCOUGH.—According to Dr. Juaritz (Siglo Medico), a physician being seized with a serious hiccough, while convalescing from gastric fever, drank in mistake an infusion of mustard, and obtained relief.

SUPPRESSION OF URINE.—The following method of restoring the secretion of urine when suppressed may be termed heroic, but has, nevertheless, proven highly beneficial, in appropriate cases—resisting every other treatment:

| | |
|---|---|
| ℞—Tr. digitalis, . . . | ℥ i. |
| Linseed meal, . . . | Q. S.—m. |

And make large poultice to be applied to the abdomen. The poultice must be made warm and the digitalis added. Let the poultice remain until the pulse falls—this is the criterion. If the pulse is high, bring it to 60 beats per minute; if low, reduce it to 40 to 50 beats. The urine will then begin to flow, but the patient must be closely watched. The remedy is to be used only in obstinate cases, and when other remedies have failed.

Dropsey.—The following diuretic combination will, in the majority of cases of dropsey, excite the kidneys to action promptly and efficiently; and while it is not "a specific," will be found of great service in procuring comfort and in giving relief to the patient:

℞—Hyd. chlo. mit. . . . gr xii.

Pulv. digital, . . . gr. xv.

Nitrate pottassæ, . . . ʒ ii.

Pulv. scillæ, . . . gr. xxiv.—m.

ft. chts. No. xxiv.

S.—One powder every four hours.

In Colliquative Perspirition—Night Sweats—of Phthisis the following is recommended:

℞—Oxide Zinc.,

Ext. Hyosciami, aa. . . gr. iv.—m.

ft. pil. No ii.

To be taken at bed-time.

Hypochondriasis, or depression of spirits, induced by dispepsia, &c., unattended with organic disease, will often be benefitted by the following:

℞—Chloroformi,

Tr. ginger, aa. . . . . . ℥ ss.

Spt. ammonia, aromat, . . ʒ ii.—m.

S.—Twenty five drops, to be taken three times a day, in a wine-glassful of milk.

LIQUID OXYSULPHTE OF IRON.

℞—Ferri sulphas . . . . . ʒ ij.

Acid nitratis . . . . ʒ iii.

Aquae distil . . . . ℥ iss.

Mix by rubbing the sulphate with the acid slowly in a mortar; gradually add the water after the sulphate is all dissolved, and filter through paper. Dose, 6 to 12 drops in water; quassæ infusion or syrup. This is regarded, by many, as the most palatable of all the iron preparations.

Prurigo.—The following prescription is said to be infalible:

℞—Ungt. citrin . . . . 3 parts.
Lard . . . . . . . 60 parts.—M.

S.—Spread a thin coating over diseased parts.

For Chancres, Ulcers, &c.—℞ Iodoform, q. s. S.—Apply to diseased parts with camel-hair pencil. Used topically it is highly recommended for cancer uteri.

Syphillitic White Swelling.—" There is a form of white-swelling of the knee, which I wish particularly to call your attention to, as the distinguishing it from others is absolutely necessary to successful treatment, for its nature is totally different from all others. It is this: patients who have secondary syphilis will often get a pain and effusion into the knee, like the white-swelling of children, and it is a true and perfect symptom of the venereal disease in the secondary form. A pupil of mine saw a case of this kind in a hospital in London, and mentioned to Sir A. Cooper what he thought it was. Sir Astley said he was mistaken, but that to satisfy him he would give the patient mercury—he did so—the knee got well under it—and Sir Astley himself declared it was the first case of the kind he had ever seen. This case may be distinguished from common white-swelling by one remarkable symptom—viz., the popliteal space is not filled up, as it always is in white swelling. In the acute cases of white-swelling, you will cure nine cases out of ten by throwing in calomel as quickly as possible."—*Medico-Chirurgical Review.*

Note.—In this age, when syphilis is more common than formerly, the physician will find ti an integra lpart of many ailments and he should direct his inquiries in such a manner as to satisfy his mind as to the fact, if such exists, of syphilitical complications, when suitable remedies should be given, if they occur.

Carbonate of Iron in Menorrhagia, &c.—Dr. Malherbe observes, that believing in the emmenagogue character usually attributed to preparations of iron, he had never ventured to use them in uterine discharges, until he had perceived a case of menorrhagia occurring in a chlorotic girl, and advantageously treated by chocolate-iron, recorded in Trousseau and Piodon's Treatise of Therapeutics. Since that time, he has repeatedly used the subcarbonate of iron with the best effects. He selects for description thirteen cases of menorrhagia, occurring in women of very different ages and temperaments, and in whom the only symptom in common was an abundant loss of blood at regular or irregular menstrual periods. Acute, as well as chronic cases yielded to the subcarbonate, which was given in doses of from ½ to 1 ℈ at varying periods of frequency. He concludes, " That the subcarbonate of iron is not an emmenagogue, but on the contrary, arrests or moderates the menstrual flux, arrests uterine hemorrhages, and is in fact one of the most powerful hæmostatic means we have at our disposal.—*Journal des Connaissances.*

Lumbago.—If there is no rheumatic or gouty condition, instant relief is sometims attainable by the spray producer—using ether or rhigoline.

LIME IN ACUTE RHEUMATISM.—The following formula is favorably recommended by those who have tried it:

℞—Unslacked lime, . . . . ℥ ii.

Sugar, . . . . . . ℥ viij.

Mix well in a mortar, then pour upon it a wine pint of boiling water; add boiling water to make up the pint and filter. Dose, 10, 20, 30, or even 40 drops, every 2 hours, in syrup.

CHRONIC CHILLS.

℞—Quin. sulph. . . . . . ʒ i.

Ferri reduct, . . . . . ʒ iss.

Strychniæ

Acid arsenosi, aa. . . . gr. iii.

Muc. g. acac. . . . . . q. s. m.

Make 60 pills.

POLYPUS OF THE NOSE.—℞ Tannin, q. s. Used as snuff and drawn up the nose through a quill. It cures. Should be used to prevent return after operation.

OPACITY OF CORNEA.—℞ Sulphate sodæ gr. ii. This is applied to the upper lid (everted) with camel-hair pencil; to be repeated daily. If reaction should be too great, mix the sulphate with a little starch—or it may be applied in solution—5 grains to ℥ iv. of water.

HEMORRHOIDS.—Dr. Ed. Young says the following ointment is unsurpassed:

℞—Pulv. matico (leaves), . . ℥ iii.
Pulv. opii. . . . . . gr. iii.
Fresh lard, . . . . . ℥ i.—m.

S.—Apply night and morning.

TONSILLITIS.—The following plan of treating this disease is regarded, by its originator, as almost a specific:

℞—Bicarb. potass. . . . . . ℈ i.

Tr. guaiac, . . . . . . ʒ ss.

(or pulv. guaiac), . . . . . gr. x.

Muc. g. accacae,

Water,aa. . . . . . . q. s.

To make a one ounce mixture.

℞—Cit. acid, . . . . . . gr. xv.

Make three powders.

S.—Make effervescent mixture, and take three times a day.

℞—Tr. iodine, . . . . . . gtt. xx.
Water, . . . . . . ℥ i.—m.

S.—As a gargle.

Colic in Infants.—The following formulæ will be found valuable in the colic of infants:

℞—Pulv. rhei.,

Fennel seed, aa. . . . ℨ ii.

Water, . . . . O. i.

Boil until ⅓ is dissipated. Dose, half a teaspoonful to half a tablespoonful, two or three times a day. It is useful in those cases attended by constipation. The addition of bromide pottassium in minute doses will, in some cases, be found beneficial:

℞—Tr. opii. . . . . . gtt. 80.
Anise oil, . . . . . gtt. 18.
Alcohol, . . . . . ℥ i.—m.

Then put in ℥ viii. of boiling water, first adding fine sugar, q. s., to suit the taste. Dose, half teaspoonful or more, according to age. This is an excellent mixture, and is highly recommended by Dr. R. A. T. Ridley, of LaGrange, Ga.

Indolent Ulcers and Suppurative Surfaces.—Maissonneuve and Grislin recommend:

℞—Charpie dipped in

Carbolic acid, . . . . . ℨ ss.

Spirits, . . . . . ℥ i.

Water, . . . . . ℥ xv.—m.

A similar treatment answers for cancer and fetid discharges of all kinds. Dr. Wolfe prefers the following, in order to get rid of the offensive smell of the acid.

℞—Soft cotton, . . . . Q. S.
Saturate with carbolic acid —.

Press the cotton of excess of acid; then dry. This is to be applied to the diseased parts. The cotton thus prepared should be kept closely in a tin box. He speaks highly of it.

Excellent Effervescing Iron Powder.

℞—Bicarb sodæ, . . . gr. xv.

Tartaric acid, . . . gr. x.

Ferri sulph. (dried), . . gr. i. to v.

Pulv. alb. sach. . . . ℨ ss.—m.

Keep in a dry place. S.—Dissolve in half wineglass of water and take while effervescing.

*New Treatment for Sprains.*—Plunge the part in water as hot as can be borne, and keep up, by adding hot water, for two hours. Then wipe limb dry, and apply adhesive strips from toes to the middle of the calf of the leg. It will give some pain. Next day reapply—again in two more days, and so on.

